# Much Ado About Nothing? The Role of Land-Based Gambling Venue Employees in Facilitating Problem Gambling Harm Reduction and Help-Seeking

**DOI:** 10.1007/s10899-023-10226-x

**Published:** 2023-06-21

**Authors:** Ben J. Riley, Sharon Lawn, Beth R. Crisp, Malcolm Battersby

**Affiliations:** 1 College of Medicine and Public Health, Flinders University, Adelaide, SA Australia; 2 Faculty of Health, School of Health & Soc. Dev., Deakin University, Geelong, VIC Australia

**Keywords:** Problem gambling, Responsible gambling, Harm minimisation, Staff, Public health

## Abstract

Over the past decade, greater emphasis has been placed on the role of the land-based gambling industry to respond to problem gambling behaviour in their venues. Despite this, there is a lack of clear information advising best practice responses by gambling venue employees. This article reviews strategies, practices, and policies employed by land-based gambling venues concerning their employees’ role in preventing gambling-related harm and responding to problem gambling behaviours. A systematic search strategy was applied to source peer-reviewed literature which identified 49 articles. The synthesised results were arranged and presented across five categories: (1) the identification of gamblers with potential problems in the venue; (2) gambling venue staff responses to gamblers with potential problems; (3) gamblers’ perspectives around venue responsibilities and interactions with gamblers with potential problems; (4) corporate social responsibility programs and the identification of gamblers with problems in the venue; and (5) gambling venue staff needs. The results suggest that most activity performed by venue staff concerning their response to problem gambling is limited to observing and documenting risky behaviours and then discussing this internally with other venue staff. Action which moves beyond this, such as approaching and interacting with identified gamblers of concern, rarely occurs. The results of this review suggest that a focus on the identification and intervention specifically with identified gamblers of concern is a particularly unhelpful aspect of the role of venue staff. The results also indicate that a re-thinking of the role frontline staff play in addressing problem gambling is necessary.

## Introduction

Over the past decade, greater emphasis has been placed on the role of the land-based gambling industry to respond to problem gambling behaviour in their venues. Gambling venue staff can serve as an important interface in communicating responsible gambling information and strategies to gamblers (Dawson & Abbott, 2011; Ponting et al., 2016; Quilty et al., 2015). There has been particular interest in the degree to which gambling venue staff can identify patrons of concern and take an active role in intervening before further harm is endured. Though several studies have investigated whether it is possible for gaming room staff to reliably identify patrons with potential gambling problems (Blaszczynski, 2002; Delfabbro et al., 2012a, 2012b; Schellink & Schrans, 2004), information advising of best practice responses by gambling venue employees once such identification has occurred, is scarce.

### Gaming Venue Employees, an Opportunity to Facilitate Help-Seeking

Given the general reluctance of individuals with gambling problems to seek help (Evans & Delfabbro, 2005; Gainsbury et al., 2014; Suurvali et al., 2009; Tavares et al., 2002) and reported low levels of awareness of help services among affected gamblers (Gainsbury et al., 2014; Hing & Nuske, 2011a), gambling venues may provide a valuable opportunity to inform patrons of available help services. This may be by way of staff interaction with patrons, or in a passive form such as signage, and pamphlets and resources in the venue. Consequently, gaming room resources and staff interactions with patrons have important public health implications. The following review will focus on staff interaction with patrons, given venue staff are among the first point of contact for individuals looking for help with gambling problems (Productivity Commission, 2010). Frontline land-based gambling venue staff, therefore, provide an important gateway to encourage patrons of concern to seek treatment and to facilitate referrals (Hing & Nuske, 2011a). Despite this, there is relatively little literature examining the policies, practices, and behaviours of venue staff’s responses to problem gambling behaviour in venues. The term ‘land-based venues’ is used to distinguish between gambling that occurs in a physical place of business as opposed to online gambling via the internet.

### Previous Literature Reviews

Five previous literature reviews published between 2014 and 2020 have reviewed the literature on approaches to harm reduction in land-based gambling venues that included content about the role of venue staff: four peer-reviewed articles (Beckett et al., 2020a, 2020b; Ladouceur et al., 2017; Livingstone et al., 2014; Skarupova et al., 2020) and one technical report (Blaszczynski et al., 2014). While other reviews of gambling operators’ harm reduction strategies, such as Tanner et al. (2017) and Forsström et al. (2021), examined consumer protection measures the role of venue staff was not considered. None of the five previous reviews that included information about the role of venue staff has focused specifically on land-based gambling venue staff involvement in problem gambling harm reduction and interactions with gamblers. These reviews will be discussed in the following paragraphs in terms of their findings and research gaps, ahead of an outline of the aims of the current review.

Livingstone et al. (2014) conducted a review of the evidence base on articles published between 1992 and 2013 of eight identified land-based venue strategies: self-exclusion, signage, messages on electronic gaming machine (EGM) screens, staff identification and interaction with gamblers, Smart-card and pre-commitment technology, removal of automatic teller machines (ATMs) from gambling venues, responsible gambling codes, and reduction in maximum bets. Only empirical studies were included from both peer-reviewed and grey literature sources. Self-exclusion programs and the removal of ATMs from gambling venues were reported to contain a ‘modest’ level of evidence. Concerning the identification of and subsequent interaction with gamblers with potential problems by venue staff, Livingston et al. (2014) concluded there was little evidence for the effectiveness of harm reduction practices where venue staff identify and interact with gamblers of concern. Moreover, based on the literature reviewed, the authors found little evidence to support the likelihood of staff correctly identifying and intervening in problem gambling behaviours, and there were some indications that once identified, venue staff may be reluctant to intervene.

Skarupova et al. (2020) conducted a review of strategies implemented by online and land-based gambling operators concerning the identification of and early intervention for gambling problems. Articles were included if they contained a systematic description of onsite identification of or intervention with gamblers with potential problems. The search was performed between September and November 2015 which yielded 67 articles. Concerning problem gambling identification and early intervention in land-based environments, the review reported that strategies mostly involved venue staff monitoring for behavioural indicators such as emotional reactions to play (e.g., irritability, complaining, crying), gambling habits (e.g., long sessions, no breaks, frequent automatic teller machine withdrawals), and social activity (sitting along, avoids company, neglected appearance). Skarupova et al. (2020) concluded that despite the availability of a broad range of known indicators for the potential identification of gamblers of concern in the venue, several obstacles hindered a proactive approach by staff. These included short-term inconsistent monitoring due to varied shifts and a lack of staff confidence in making uninvited approaches related to harm minimisation. Skarupova et al. (2020) highlighted the lack of sufficient evidence about the effectiveness of both online and land-based venue harm reduction strategies.

Beckett et al. (2020a, 2020b) conducted a systematic review of 17 articles, all empirical studies, published between 2001 and 2018 that investigated the effectiveness of responsible gambling training for land-based gambling venue staff. Many jurisdictions with legalised gambling require gambling operators to participate in responsible gambling training, such as Canada (Quilty et al., 2015) and Australia (Roberts, 2008). Accredited responsible gambling training is mandated in most states and territories in Australia (South Australia [SA], Australian Capital Territory [ACT], New South Wales and Tasmania), and actively encouraged in Victoria, Northern Territory and Queensland, with SA and the ACT possessing the most rigorous government regulations concerning responsible gambling codes (Roberts, 2008). For instance, SA and ACT are the only Australian jurisdictions where legislation makes it explicit that gaming room staff are required to play an active role in identifying gamblers with potential problems (Roberts, 2008). Training for gambling venue staff, therefore, is an important part of responsible gambling programs. Synthesised findings of the 17 empirical studies included in Beckett et al. (2020a, 2020b) review suggested that the programs did improve staff knowledge and confidence in managing individuals experiencing gambling harms, however, they lacked provision of practical skills for dealing with difficult situations. The results of the review suggested that training programs should pay particular attention to staff interactions with patrons of concern, especially in challenging situations. Several methodological weaknesses (e.g., lack of comparative control group and baseline data) were reported across the studies which precluded the researchers from drawing any conclusions as to the effectiveness of the training programs in reducing gambling-related harm (Beckett et al., 2020a, 2020b).

Ladouceur et al. (2017) reviewed peer-reviewed empirical evidence underpinning responsible gambling strategies and examined only studies that were conducted using in-situ gambling environments, and at least one of the following criteria: a matched control or comparison group, repeated measures, and one or more measurement scales. The review of 29 included studies highlighted five specific areas of scientific research concerning responsible gambling strategies: (1) self-exclusion; (2) gambling behaviour to develop algorithms that can identify problem gambling; (3) limit setting; (4) responsible gambling EGM features; and (5) staff training. The only area that included findings related to staff interacting with identified gamblers of concern was staff training. The review concluded that interactions between staff and gamblers around problem gambling, and the identification of gamblers with potential problems by venue staff, represented a significant challenge, and empirical research in this area remains underdeveloped. Only six of the studies included all three criteria for scientific rigour. The authors concluded that few responsible gambling strategies could be considered “best practices”.

In a non-systematic literature review, Blaszczynski et al. (2014) reviewed operator-based approaches to gambling harm minimisation for land-based and online forms of gambling. The authors suggested that the current model of responsible gambling is overly passive and not proactive. Concerning venue staff, they concluded that staff are reluctant to intervene with gamblers suspected of having problems because they feel they lack proper training to handle sensitive potentially difficult interactions. In addition, they recommended that attempts should be made to engage the player with responsible and problem gambling guidance before significant harm is experienced. The review called for adequate training for staff in responsible gambling and proposed “candid specification of staff responsibilities would increase staff self-efficacy in this context” (Blaszczynski et al., 2014, p. 80).

The five literature reviews on approaches to harm reduction in land-based gambling venues described above, (Beckett et al., 2020a, 2020b; Blaszczynski et al., 2014; Ladouceur et al., 2017; Livingstone et al., 2014; Skarupova et al., 2020) indicate overall there is little evidence that the identification of and engagement with gamblers of concern by venue staff does anything to reduce gambling-related harm. Venue staff have identified that engaging gamblers about problem gambling is a significant challenge (Ladouceur et al., 2017; Skarupova et al., 2020) as staff lack confidence (Livingstone et al., 2014; Skarupova et al., 2020) and are therefore reluctant to do so (Beckett et al., 2020a, 2020b; Blaszczynski et al., 2014). Further training of staff that focuses on the engagement of gamblers of concern was suggested as a strategy to increase staff confidence (Beckett et al., 2020a, 2020b; Blaszczynski et al., 2014).

### Aims of the Current Review

The previous reviews have focused on either, empirical studies only (Livingstone et al., 2014; Tanner et al., 2017) or both online and land-based gambling environments containing multiple forms of gambling (Skarupova et al., 2020; Tanner et al., 2017), and included literature up until 2015. The present review adds to this literature by taking a narrower focus on examining harm minimisation approaches used by employees of land-based venues which operate EGMs and taking the opportunity to update the literature. The rationale for limiting the focus of this review to casinos and land-based venues containing EGMs (i.e., not venues which only operate horseracing, sports betting, lotto, and keno) is since EGMs are among the most frequently reported form of gambling by gamblers with problems (Armstong & Carroll, 2007; Productivity Commission, 2010) and the majority of gambling expenditure in Australia emanates from casinos and EGMs (Queensland Government Statistician's Office, 2019). The following research question guided this review: what is known about the policies, practices and behaviours of land-based gambling venue staff concerning their responses to EGM problem gambling behaviours?

## Methods

In this current review, peer-reviewed literature on gambling venue employees’ role in facilitating harm minimisation and help-seeking for gamblers is reviewed. This study aims to review the policies, practices and behaviours employed by land-based gambling venues concerning their employees’ role in preventing gambling-related harm and responding to problem gambling behaviours. Articles that focused on describing or evaluating staff training were not included as this literature has been previously reviewed (Beckett et al., 2020a, 2020b). The aim of the present review is not purely to evaluate the evidence, but rather, to describe and summarise the literature and assess the evidence where available.

A systematic approach was applied to search the academic electronic databases for articles on gambling venues’ responses to problem gambling. A research librarian was consulted to assist with developing the search strategy. Four electronic databases were searched: Scopus; ProQuest; PsycInfo; Web of Science. Titles, abstracts, and keywords were searched using the following terms: “Gambl* AND (policy or policies or referral or staff or employee or “support program”) AND (gambling venue* OR casino* or hotel* or hospitalit* or “gaming room*”)”.

The search process was performed on the 24th of February 2021. The final synthesis of the findings from this review offers a review of previous research, with a specific focus on current practices concerning gambling venue employees’ role in harm minimisation and assisting affected gamblers to seek help.

### Inclusion and Exclusion Criteria for the Review

Documents were included if they were written in English and contained information about employees of land-based venues that operate EGMs, involvement with problem gambling harm minimisation and the facilitation of help-seeking. Given the expansion of legalised gambling opportunities, particularly casinos and EGMs, and a coinciding increase in problem gambling rates in Australia and North America in the early 1990s (Williams et al., 2012), alongside changes in gambling policy and legislation over this period, for instance, casinos in America and Australia began implementing responsible gambling programs and policies in the 1990s (Hing, 2003, 2010), articles published in 1990 and later were included. Table 1 presents the inclusion and exclusion criteria.

**Table 1:** Inclusion and exclusion criteria for the review

Inclusion criteria	Exclusion criteria
English language Published in 1990 or later	Documents not in English Published before 1990
Peer-reviewed journal articles	Literature review and research protocols Grey literature
The venue contains electronic gaming machines (inc. video lottery machines) and is land-based including hotels, community/sporting clubs and casinos	The focus of the venue is sports betting, horse racing or other wagering, and does not operate electronic gaming machines Online gambling
The document contains information on gambling venue employees’ response to problem gambling or the promotion of harm minimisation	The document does not address gambling venue employees’ response to problem gambling or the promotion of harm minimisation

**Fig. 1:**
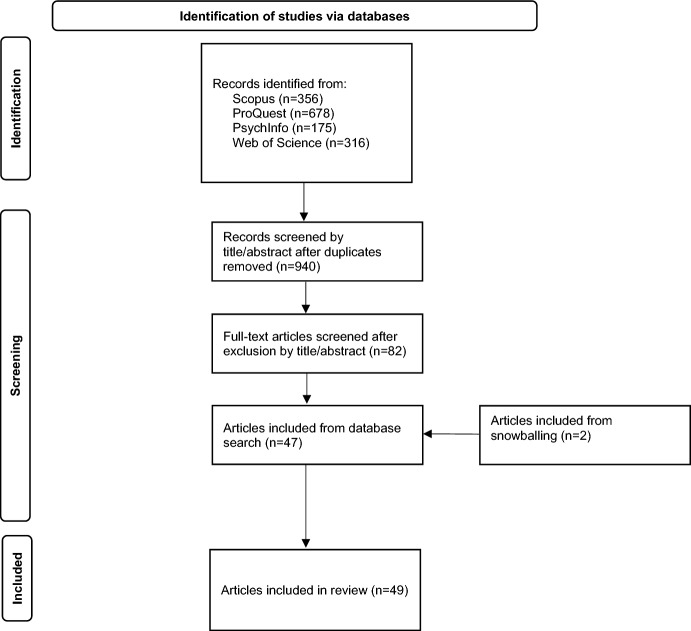
PRISMA flow chart for article selection

A total of 1525 documents were located and combined into an Endnote library for screening and duplicates removed. Inclusion criteria were then applied to the remaining 940 documents resulting in 82 documents requiring a full-text read. Backward snowballing was then employed to search the reference lists of included articles (Horsley et al., 2011) which yielded an additional 2 items resulting in 49 articles for this review. Figure 1 presents the results of this process in a PRISMA flow diagram.

The 49 articles included in the review were loaded into NVivo Qualitative Data Analysis Software (2018) for coding purposes. While it is most commonly used for qualitative data analysis, NVivo is an effective tool for performing literature reviews (Bandara et al., 2015). For coding purposes, three preselected themes were used as these were considered important and appropriate to explore to achieve the research aims: corporate social responsibility programs and the identification and response to problem gambling behaviours in the venue; the identification of gamblers with gambling problems in the venue; gambling venue staff responses to patrons of concern. During the coding process, two additional themes emerged: gamblers’ perspectives around venue responsibilities and gambling venue staff needs. The five themes along with the main findings are presented in Table 2 and are discussed in the following results section.

**Table 2:** Themes and sub-themes

Themes	Main findings
Corporate social responsibility programs and the identification and response to problem gambling behaviours in the venue	Lack of clear guidelines for staff other than documenting, outlining what action staff should take once problem gambling has been identified
The identification of gamblers with gambling problems in the venue	Despite agreement with a range of indicators, venue staff face significant challenges in making accurate identification
	Evidence is mixed concerning the accuracy and confidence of staff to identify which gamblers have problems
Gambling venue staff responses to patrons of concern	Minimal evidence exists of the effectiveness of venue staff interacting with identified gamblers to effectively achieve harm reduction
Gamblers’ perspectives on venue responsibilities and staff interactions with patrons of concern	Few studies investigating gamblers’ views on venue responsibilities. The limited evidence indicates gamblers rarely approach staff for help, and are reluctant to welcome uninvited approaches by staff
Gambling venue staff needs	Greater clarification to put responsible gambling policies into practice, and clearer procedures and direction around indicators of problem gambling and how to approach patrons of concern

## Results

### Corporate Social Responsibility Programs and the Identification and Response to Problem Gambling Behaviours in the Venue

A range of articles described and discussed gambling venues’ various codes of conduct, which outlined both mandatory and voluntary rules and responsibilities of gambling operators. Many jurisdictions’ gambling regulatory codes required gambling operators to employ a duty of care towards gamblers. Frequently this included staff identifying individuals experiencing gambling-related harm and rendering appropriate assistance. Whilst directives informing venue staff to observe and identify potential problem gambling behaviours among their patrons were common across the literature, much less common were clear guidelines outlining what action staff should take once risky gambling was identified.

The most frequently described advice was that frontline staff should document observed risky behaviours and then report them to a senior staff member or a staff member dedicated to the responsibility of managing problem gambling issues (Beckett et al., 2020a, 2020b; Breen et al., 2006; Guerrier & Bohane, 2013; Hancock & Hao, 2016; Meyer et al., 2009; Thompson, 2007).

Several jurisdictions had more formal programs to identify and respond quickly to gamblers with potential problems. In Switzerland, all casinos must implement preventative measures, for instance, the early detection of patrons displaying signs of potential gambling problems. Staff must observe and prepare a report if they witness signs of problem gambling, which is examined by a supervisor. If specific risky behaviours are involved, the supervisor must approach the player concerned immediately (Thompson, 2007). In Germany, casino employees are required by law to impose forced exclusion on individuals at risk of problem gambling (Kotter et al., 2018). Similarly, Swiss casinos have the mandate to impose involuntary exclusion orders on gamblers they believe are gambling beyond their means, and the exclusion orders are applied to all casinos across the country (Delfabbro et al., 2007; Lischer & Schwarz, 2018).

While the information gained via frontline staff observation was not typically recorded in any formal documentation system, some Canadian jurisdictions did report documenting and collating observable signs of problem gambling among players. For example, several Canadian casinos use an electronic monitoring system ‘iCare’ (intelligent player care program; (Davies, 2007). The iCare system can capture and interpret data from the operator’s casino management system and identify patrons deemed to be at risk. While the authors state that player tracking data is used to identify those deemed to be at various levels of risk, no detail is provided as to what criteria are used to make these assessments. Once a patron of concern is identified, the system is enabled to notify operators when the patron is in the casino and provide staff with information about their behaviour and clear processes to follow (Davies, 2007). A dedicated onsite responsible gambling centre in a United States casino was described (Gray et al., 2020a, 2020b) where frontline staff observe and document gamblers’ behaviours and engage with those displaying signs of risk. While staff generally had positive views of the program, two-thirds reported they had not had contact with the onsite centre or its staff (Gray et al., 2020a, 2020b).

Across the literature, studies examining the effectiveness of responsible gambling programs were lacking, however, there has been some research that has examined the implementation and effectiveness of responsible gambling regulatory codes. Breen et al. (2005) investigated the levels of implementation of the Queensland Responsible Gambling Code of Practice in Australia and found lower implementation rates among smaller venues than those with larger gambling installations. Impediments included high staff turnover and remote locations, whilst facilitators included staff training and education (Breen & Hing, 2007), regular audits, and elements of the Code that overlapped with legal obligations for the gaming operator (Breen et al., 2005). Fiedler et al. (2020) conducted a recent review of seven responsible gambling programs of land-based EGM operators in Germany. In addition to reviewing responsible gambling programs, the researchers surveyed 512 gamblers in treatment to examine their experience of the described responsible gambling initiatives. The results of their study indicated that the responsible gambling programs reviewed contained a range of measures, most of which were mandatory except notably, those that involved staff approaching patrons displaying problem gambling behaviours, to help them. Moreover, the results of their survey revealed that almost half the gamblers reported they felt their significant losses were noticed by staff, with around a third believing that staff were aware of their problem. However, only 1% of gamblers with problems reported being approached by staff and referred to support services (Fiedler et al., 2020).

### The Identification of Gamblers with Gambling Problems in the Venue

Research into the feasibility of venue staff identifying individuals gambling at harmful levels through observation indicates that while there appears to be a reliable set of behavioural indicators, using them effectively in practice presents several challenges. The concern around gambling operators diagnosing individuals experiencing gambling-related harms has been raised by leading problem gambling researchers (Blaszczynski et al., 2004), in particular, the use of relying solely on observable indicators (Delfabbro et al., 2016; Hing et al., 2013) and appears to have been reflected in the handful of studies that have endeavoured to empirically investigate if it is possible to identify individuals with gambling problems in the venue.

A study in Nova Scotia (Schellink & Schrans, 2004) investigated combinations of potential indicators that could be used to identify individuals with gambling problems with a high degree of confidence. A wide range of visible and non-visible cues, when occurring in combination during a visit, was found to have a high confidence value (90% or better) in identifying if someone had a gambling problem. The authors, however, made the point that the relatively low frequency that behavioural indicators are likely to occur, at the precise time a single onlooker might observe them, poses significant challenges for effectively using such indicators in practice. In South Australia, Delfabbro et al., (2012a, 2012b) investigated the accuracy of gambling venue staff in identifying gamblers with problems in venues. The study found that venue staff were able to identify only 36% of patrons experiencing problems with gambling. Many gamblers who self-reported at least moderate gambling problems, were not classified as having any problems by staff. On the other hand, there were gamblers in the ‘no risk’ category classified by staff as having gambling problems. In another study, a computer-based model using an algorithm to decode observations made by trained staff about casino gamblers was developed by Ifrim (2015). When the model was run with simulated groups of gamblers both with and without problems, it was able to identify correctly in about 95% of the cases.

The outcomes of these studies are consistent with the conclusions provided by Schellinck & Schrans (2004) and Allcock (2002) in that, although it is theoretically possible to identify individuals with gambling problems using a range of behavioural indicators, there are many challenges facing venue staff if they are to rely on such indicators in practice. Delfabbro et al., (2012a, 2012b) argued that while staff may be able to observe potential indicators, this would require a period of continuous observation that would likely be impractical for staff to perform given their other competing duties. The authors recommended that venues use multiple indicators, including observable and electronic monitoring, and consolidate information about individual patrons across multiple observers and periods of observation (Delfabbro et al., 2012a, 2012b). This is consistent with Hancock et al. (2008) who recommended that clear protocols be developed to monitor individual patrons’ indicators over time and that electronic player tracking technology should be adopted.

Along with the feasibility and accuracy of using observable indicators to detect problem gambling, venue staff’s confidence in using such tools has been investigated. In general, it has been reported that staff are aware of the important elements of the definition of problem gambling and the indicators (O'Mahony & Ohtsuka, 2015), with one study reporting that collectively staff were able to identify 22 potential indicators of problem gambling (Hing & Nuske, 2011b). Gambling help counsellors have likewise indicated they believe venue staff can identify patrons of concern (Hing & Nuske, 2011a). Although the majority of venue staff involved in these studies indicated they could recognise a patron with a gambling problem (Hing & Nuske, 2011a, 2011b; Hing et al., 2013; O'Mahony & Ohtsuka, 2015), some of the challenges reported included that competing work demands meant staff were not able to observe patrons all the time (Hing et al., 2013). In addition, staff were concerned about the potential to mistakenly identify a regular gambler as a patron of concern, where there was no problem (false positives) (Hing et al., 2013).

Other research has investigated differences in observable behavioural indicators among diverse groups of gamblers. Delfabbro et al. (2018) examined differences between male and female gamblers. Signs of emotional distress were more commonly reported by female gamblers with problems, while males were more likely to display aggression towards others in the venue and the gambling machines (Delfabbro et al., 2018). O’Mahony & Ohtsuka (2015) examined how venue staff identified and perceived diverse groups of gamblers (age, gender, and specific cultural groups) concerning displaying signs of problem gambling. Staff displayed more empathy towards older gamblers, classifying them as pensioners who could not afford to lose money, and they were more sympathetic towards women, particularly if they became upset and cried. However, they were unsympathetic towards young males whom they perceived as loud and aggressive and who could afford to lose large amounts of money (O'Mahony & Ohtsuka, 2015). This is concerning given young men are particularly vulnerable to developing gambling problems (Riley et al., 2021).

In summary, the literature suggests that despite general agreement concerning a range of reliable and observable indicators to detect individuals with gambling problems in situ (Delfabbro et al., 2012a, 2012b), venue staff face significant challenges in making accurate identification of which gamblers have problems, with some researchers proposing that this is more challenging than recognising alcohol impairment in the alcohol context (Hancock et al., 2008). The results of the studies reviewed indicate that the accuracy and confidence of gambling venue staff in identifying gamblers with problems are mixed. More importantly, effective identification of individuals experiencing gambling-related harms will only be useful if it leads to appropriate and effective action (Hancock, 2011). This sentiment was also offered by Livingston et al., (2019) who concluded that, while it may be possible for venue staff to identify such gamblers in situ, gambling operators must be required to implement mandated interventions once identification occurs.

### Gambling Venue Staff Responses to Patrons of Concern

Responding to patrons with gambling problems in the venue can be differentiated by two distinct scenarios: a patron approaching a staff member for assistance about their excessive gambling (invited contact); and a staff member initiating contact with an identified patron of concern about their gambling (uninvited contact). Regarding invited contact, this appears to be an exceedingly rare occurrence (Hing & Nuske, 2011b, 2012a) with some staff reporting never having been approached (Hing & Nuske, 2011b) and when gamblers do approach staff is it mostly to seek assistance with self-exclusion programs (Hing & Nuske, 2011b).

Although staff have generally reported feeling comfortable and confident in responding to patrons of concern who do initiate contact (Hing & Nuske, 2011b, 2012a), they have conveyed discomfort and apprehension dependent on the patrons’ perceived level of embarrassment (Hing & Nuske, 2012a). When asked about situations involving being approached by a family member or friend expressing concern about a patron’s gambling, staff were much less confident, which was due to their concerns around not wanting to breach patrons’ privacy (Hing & Nuske, 2012a) and a lack of knowledge and clear procedures (Hing & Nuske, 2011b).

A study by Quilty et al. (2015) reported that, although staff found that a range of observable signs was useful in identifying patrons with gambling problems, responding to such concerns was viewed as a challenge, and staff indicated they were forbidden by management to approach patrons of concern (Quilty et al., 2015). An Australian study involving interviews and focus groups with 40 gamblers and 20 professionals (Rintoul et al., 2017) revealed only minimal evidence of harm minimisation interactions between venue staff and gamblers. The results suggested that signs of problem gambling are normalised in venues operating EGMs and that venues often failed to respond to signs of problem gambling, in fact, encouraged it (Rintoul et al., 2017).

Studies that examined venue staff behaviour around initiating uninvited contact with identified patrons of concern, indicated that overwhelmingly, staff reported general unease and reluctance (Hing & Nuske, 2012a, 2011a, 2011b; Hing et al., 2013; Riley et al., 2018). Hayer et al. (2020) analysed an administrative data set of excluded gamblers from land-based gambling venues and examined the compliance of staff in implementing various gambler protection measures. Results indicated that staff provided appropriate interventions to players with signs of problematic gambling, in only 7% of cases. A qualitative study by Riley et al. (2018) examined the experiences of both venue staff and EGM gamblers concerning the identification of and staff responses to problem gambling behaviours in situ. Both staff and gamblers described an awareness of a conflict between staff facilitating the use of EGMs in the context of a commercial business while monitoring patrons for potential problems. Such conflict influenced interactions between the two: gamblers were reluctant to seek assistance from staff, and staff were reluctant to approach gamblers displaying problems. A major barrier identified by frontline staff concerned their fears of the potential consequences of misidentifying and or causing distress to a gambler perceived as at risk. Such fears are recognised by some experts who have argued that “unjustified intrusion is likely not the way to promote responsible gambling” (Blaszczynski et al., 2004, p. 312). The Riley et al., (2018) study suggested that gambling venue staff should disseminate harm reduction information to all gamblers, with less emphasis on identifying individual gamblers with potential problems.

The studies reviewed concerning venue staff interactions with gamblers identified as potentially having a problem revealed several factors that have contributed to apprehension among staff. These included a lack of prescribed procedures about how to initiate uninvited contact (Hing & Nuske, 2011b), feeling ill-equipped and fearful of a negative reaction (Hing & Nuske, 2011a) in particular an angry or even violent reaction from patrons, and concerns around invading patrons’ privacy (Hing & Nuske, 2012a). One study reported that female staff were more reluctant to intervene with identified gamblers of concern due to feared potential negative reactions from the gambler (Tomei & Zumwald, 2017). However, while uninvited contact with patrons of concern occurs infrequently (Hing & Nuske, 2011b; Hing et al., 2013; Rintoul et al., 2017), there was some evidence that when it did occur it could potentially be effective and lead to interventions such as self-exclusion and or referral to gambling help services (Hing & Nuske, 2011b, 2012a).

### Gamblers’ Perspectives on Venue Responsibilities and Staff Interactions with Patrons of Concern

Information concerning gamblers’ perspectives on venue staff’s role in problem gambling harm minimisation in the venue was scarce, and the results from studies that did examine gamblers’ perspectives on the venues’ responsibility to protect players were mixed. In a study of individuals with gambling problems who played EGMs and who had participated in a self-exclusion program, Hing and Nuske (2012b) reported that most respondents believed it was up to them to manage their problem and disagreed it was the venues’ responsibility. There was some indication that gamblers’ views regarding whether venue employees should do more to protect patrons from gambling related harm, were influenced by the gamblers’ level of risk. In a sample of almost 5000 regular casino gamblers in the United States, Gray et al. (2021) found that gamblers who scored positively on a brief problem gambling screening tool were more likely to view other stakeholders (e.g., casino employees, government regulators, public health officials) responsible for addressing gambling-related harm than those who scored negatively. Similarly, a study involving interviews with 348 casino gamblers reported those with gambling problems were more likely to hold the casino responsible for harm reduction than those without problems (Prentice & Woodside, 2013), consistent with the findings of a study of 3748 casino loyalty card holders (Gray et al., 2019).

While Jackson et al. (2015) investigated land-based EGM gamblers’ perspectives on harm minimisation measures, venue staff’s role and interaction with venue staff were not addressed. Hing (2004, 2005) conducted a study investigating gamblers’ perspectives on the efficacy of responsible gambling measures in New South Wales clubs. Although the quantitative findings did not address interactions between venue staff and gamblers (Hing, 2004), the qualitative findings reported some gamblers advocated for greater intervention by staff, for example, making them aware of the time spent gambling if they have been there for an extended period, or inviting them to discuss the extent of their gambling (Hing, 2005). Riley et al. (2018), however, reported that EGM gamblers indicated that any harm reduction approach by venue staff could be perceived as hypocritical and disingenuous due to a perceived conflict of roles, and therefore unwelcomed.

### Gambling Venue Staff Needs

The acknowledgement of problem gambling as an important public health issue (Latvala et al., 2019; Productivity Commission, 2010; The Lancet, 2017) has placed increased pressure on gambling venues to address their responsible gambling practices and provide a safer environment to gamble, which had given rise to several unique challenges for venue staff (Hing & Nuske, 2012a). Given gaming room staff play an important role in problem gambling harm reduction, it is necessary to understand what is known about the challenges and stressors that are unique to gaming room employees. Several challenges and needs have been highlighted in the literature.

Gaming room managers indicated that although they are aware of a variety of information sources on government policy and processes, they would benefit from assistance with greater clarification to help inform their responsible gambling practices (Breen, 2005). Frontline staff also expressed the need for clearer procedures and direction around indicators of problem gambling and how to approach patrons of concern (Hing & Nuske, 2011b). This was supported by Hing et al. (2013) who point out that the Queensland responsible gambling code does not provide clear advice concerning how to determine if a patron has a gambling problem and how to respond. While gamblers rarely approach venue staff for assistance, when they do it is typically to assist with self-exclusion programs (Hing & Nuske, 2011b). However, the need for venue staff to be better equipped to facilitate self-exclusion programs was also highlighted (Motka et al., 2018; Pickering et al., 2019).

Another challenge expressed by venue staff concerned with coping with increased negative emotional responses by patrons, especially anger and distress (Tiyce et al., 2013). In addition, staff have identified role conflict and role ambiguity as a source of stress (Riley et al., 2018; Tiyce et al., 2013) given that on the one hand, they have the role of attracting patrons, while at the same time, there is an expectation that they approach patrons of concern, which may ultimately lead to driving the patron away to another hotel (Hing & Nuske, 2012a). Staff described a conflict between their dual roles of facilitating the use of gambling machines in the context of a commercial business and their concurrent role concerning minimising gambling-related harm to venue patrons. This perceived conflict left them feeling uncertain about how they should act towards gamblers displaying signs of problem gambling; their role was ambiguous. Underpinning the conflict was an ethical, moral conflict of earning their living from working in the venue yet seeing the harm arising from excessive gambling for some patrons, which was experienced by staff as particularly stressful.

Exposure to players exhibiting signs of problem gambling and challenges responding to them was related to lower job satisfaction among venue staff (Quilty et al., 2015). Corporate social responsibility and an expectation of effective responsible gambling strategies were found to positively influence employees’ organisational commitment and job satisfaction (Song et al., 2015). In a study of 2192 casino employees, Abarbane et al. (2019, 2020) observed that venue staff’s perceptions of the effectiveness of responsible gambling programs increased with their degree of contact with gamblers. Overall, the literature indicated there is a need for staff to be better equipped to sensitively respond to signs of problem gambling (Beckett et al., 2020a, 2020b; Hing & Nuske, 2011b, 2012a; Oehler et al., 2017; Quilty et al., 2015).

## Discussion

The evidence concerning gambling venue employees’ confidence in their ability to detect individuals with gambling problems is mixed. Overall, staff reported confidence in their ability to identify observable signs of problem gambling and less confidence in detecting which gamblers had problems. That is, being able to identify observable indicators and then judging a patron as having a gambling problem, are not the same thing. The limited research in this area suggests that in practice, staff are poor at making accurate identifications of gamblers with problems.

In general, staff reported confidence in responding to patrons who initiate engagement and ask for assistance with a gambling-related problem. However, the evidence suggests that patrons rarely approach staff for assistance with a gambling problem, and staff are much less confident in responding to problem gambling behaviour by initiating uninvited contact (Beckett et al., 2020a, 2020b), and have less empathetic views of young male gamblers displaying signs of problematic gambling (O'Mahony & Ohtsuka, 2015). A reluctance of initiating uninvited contact with the aim of problem gambling harm reduction is largely due to fear of a negative response from patrons (Hing & Nuske, 2012a; Riley et al., 2018). Responsible gambling training programs appear to be effective at increasing staff knowledge about gambling and responding to problem gambling behaviours (Beckett et al., 2020a, 2020b), though would benefit from more detailed practical skills-based information about making sensitive responses to gamblers identified as potentially having a problem (Quilty et al., 2015).

The results of the current review are consistent with previous reviews on this topic in that overall, there is little evidence that the identification of and engagement with gamblers of concern by venue staff does anything to reduce gambling-related harm (Beckett et al., 2020a, 2020b; Blaszczynski et al., 2014; Ladouceur et al., 2017; Livingstone et al., 2014; Skarupova et al., 2020). An important topic addressed by the current review, absent in previous reviews, concerns gamblers’ perspectives on venue responsibilities and staff interactions with patrons of concern. There is a lack of research concerning gamblers’ perspectives on venue staff’s role in problem gambling harm minimisation in the venue, and the results from studies that addressed this topic were mixed. The current review has identified an important area that has been largely overlooked by researchers, the perspectives of individuals with lived experience. We found little evidence that gambling harm minimisation policies have been informed by lived experience experts. There is growing recognition of the value of persons with lived experience as advocates, through contributing their unique expertise, and their ability to provide high-level and practical solutions, and make a meaningful influence on policy and practice (Sunkel & Sartor, 2022).

From the literature reviewed, most of the activity performed by venue staff concerning responding to problem gambling involves observing and documenting risky behaviours and then discussing this internally with other venue staff. Action which moves beyond this, such as approaching and interacting with identified gamblers of concern, rarely occurs. As previously noted by Hancock (2011), identifying individuals experiencing gambling-related harm is of little use if it does not lead to effective harm reduction action. This is an important point as the widespread activity performed by gambling operators around observing and documenting potentially risky gambling behaviours and delivering mandated staff training programs, can lead to an impression that there is sufficient harm minimisation activity occurring across the gambling industry. Whereas the evidence suggests there is very little direct harm minimisation activity occurring in venues between staff and individuals who do experience gambling problems, and the little action that does take place is almost entirely targeted towards those with obvious and significant issues who display disruptive behaviours such as aggression or violence (Hing et al., 2013; Riley et al., 2018). Further, male gamblers, who are among the highest risk of developing problems, particularly young males (Riley et al., 2021), appear to be the least likely to receive harm minimisation interactions by venue staff (O'Mahony & Ohtsuka, 2015). There were some exceptions to this, such as the more proactive mandated responses by casino staff in Switzerland (Thompson, 2007) and Germany (Kotter et al., 2018).

Gaming room employee stress resulting from role conflict is itself gambling-related harm that has largely been overlooked by researchers. High staff turnover within the hospitality industry is a well-documented issue and is largely attributable to work pressures and stress (AlBattat et al., 2014; Dwesini, 2019). Moreover, employee turnover has been reported to increase as employees’ roles become more specialised and staff require more training (Pranoto, 2011), therefore, it is unsurprising that staff turnover and employee stress are of particular concern in gambling workplaces (Breen et al., 2012). Role conflict within gaming rooms, which is in part driven by current responsible gambling policies, is an important contributor to employee stress.

In addition to contributing to employee stress, this review has found that role conflict impedes staff from promoting help-seeking among gamblers of concern and, in effect, carrying out their responsible gambling duties. Staff described notable unease about identifying individuals with potential gambling problems, as they felt making what they described as a moral judgement against a patron conflicted with their hospitality role. Considerable ambiguity was expressed by staff regarding which gamblers should be approached, as many appeared to display signs of problem gambling. As such, role conflict was identified as a significant source of stress for venue staff: the conflict and ambiguity that resulted from their responsible gambling obligations were major contributors to workplace stress.

While there has been some discussion of role conflict among venue staff in the gambling literature (Hing & Nuske, 2012a; Riley et al., 2018; Tiyce et al., 2013) it is a significant gambling-related harm that has largely been ignored. Further, the little attention role conflict has received has focused on its relationship with employees’ stress and burnout (Hing & Nuske, 2012a; Tiyce et al., 2013) with just one study (Riley et al., 2018) that examined its impact on staff’s ability to effectively carry out their responsible gambling duties (such as facilitating help-seeking) and gamblers’ perceptions of such duties. Given the abundance of literature confirming the negative effects of role conflict and ambiguity in the workplace (e.g., (Loscocco & Roschelle, 1991; Nouri & Parker, 2020; Örtqvist & Wincent, 2006; Saks et al., 2007) and evidence of its existence among staff in the gambling industry (Hing & Nuske, 2012a; Riley et al., 2018; Tiyce et al., 2013), it is surprising that this issue has been overlooked by gambling researchers and policymakers. Instead, there appears to have been a much greater focus on developing methods to help venue staff detect gamblers with problems (e.g., observable behavioural indicators) with almost no consideration of the existence and influence of role conflict and ambiguity on staff or gamblers. A responsible gambling framework that emphasises the identification of problem gambling behaviours before action is considered (and as this review has indicated even when it is considered it rarely amounts to effective action), reinforces an assumption that the development of gambling addiction is inevitable and acceptable and completely ignores earlier opportunities for harm prevention.

The findings of this research suggest that policy initiatives such as corporate responsibility programs implemented at the gambling venue level should be properly evaluated and involve lived experience experts. Implementation of responsible gambling messages could be investigated using qualitative research methodology to examine how gamblers experience such messaging, and if it does lead them to feel less conflicted about offers of help from venue staff. In the same manner, venue staff’s experiences could be explored to examine if the presence of such messaging leads them to feel less conflicted, and thereby more confident in disseminating gambling harm prevention information as a part of their duties. There are several validated psychometric tools designed to measure role conflict and role ambiguity e.g., (Bowling et al., 2017; Siegall, 2000). To date, no study has examined the constructs, role conflict or role ambiguity, among gambling venue staff using quantitative methodology. Doing so would allow researchers to assess the prevalence and levels of role conflict and ambiguity among staff and monitor them as mitigating strategies are introduced. Researchers could then observe if a reduction of role conflict and ambiguity among venue staff leads to lower levels of stress and greater job satisfaction. Further, researchers could then examine any if a reduction in role conflict and ambiguity among staff improves their attitudes towards interacting with gamblers concerning harm minimisation and help-seeking, and ultimately if it leads to more gamblers seeking help. Overall, more attention should be paid to ensuring gambling harm reduction strategies are guided by evidence, which includes proper evaluation. Policy interventions will have little impact if they are not based on evidence (Okechukwu et al., 2014).

The inaction of gambling venues in directing their employees to take a more proactive harm minimisation response towards gamblers displaying signs of problem gambling, beyond that which involves observing and documenting, may also be a result of the largely self-regulatory nature of the various codes of conduct. Similar concerns have been raised by alcohol experts about self-regulatory measures and responsible drinking messages from the alcohol industry. As Fiedler et al. (2020) note, some studies argue that due to the alcohol industry’s conflicting objectives: to encourage consumption in the service of generating profits whilst discouraging overconsumption, the alcohol industry purposefully produces responsible drinking campaigns for marketing purposes. The authors go further in arguing that such campaigns are not only ineffective but may in themselves be harmful by reinforcing current drinking patterns (Fiedler et al., 2020).

The results of this comprehensive review indicate that the role of gambling venue staff in promoting harm reduction directly with patrons, involves strategies that are largely individually focused, and there is little evidence for their effectiveness. Further, the focus is clearly on gamblers who display overt signs of impaired control. These outcomes are unsurprising given many countries have adopted a formal, non-binding responsible gambling strategy based on the original Reno 2004 Model in the United States (Blaszczynski et al., 2004; Nature, 2018), a model largely based on individual responsibility and informed choice, which has influenced academic, policy and government regulatory debate for over a decade (Hancock & Smith, 2017a). There is growing criticism of a responsible gambling model based on the notion of individual responsibility for harm, and a call for an approach based on consumer protection, public health, and operator duty of care (Abbott, 2020; Hancock & Smith, 2017a, 2017b; The Lancet, 2017). Indeed, The *Lancet Public Health* Commission on Gambling was recently established to thoroughly consider global issues concerning gambling, including the critical appraisal of regulatory, political, and public health responses (Wardle et al., 2021).

While a move away from an individual-level narrative towards a more holistic perspective is gaining some attention, supporters of the Reno Model for responsible gambling (Blaszczynski et al., 2004) continue to argue in favour of a model which emphasises individual responsibility (Shaffer & Ladouceur, 2021; Shaffer et al., 2020). Shaffer et al. (2021) argue that “assuming personal responsibility is at the centre of personal development” (p. 1075) and go so far as to suggest that measures that encourage individuals to avoid personal responsibility and see their behaviour as a function of external pressures and influence, may impede treatment and recovery from gambling problems (Shaffer & Ladouceur, 2021). The authors go further and describe the shift away from individual responsibility as a “troubling movement” (p.1072) and make the comment that, while accepting personal responsibility can be a painful process, it is a sign of maturity and personal health.

There would be few who would disagree that with maturity comes acceptance of responsibility, however, in the context of a gambling disorder, it is an overly simplistic statement. Considering the findings of the current literature review, it is argued that an emphasis on individual autonomy as an ideal to maintain the responsible gambling status quo, is convenient for the industry and those who benefit from industry profits. This stance helps maintain revenue while promoting an image of social responsibility yet does little to prevent EGM gamblers who have developed problems, from further harm or to facilitate help-seeking. While advocating for individual responsibility in the context of problem gambling, Shaffer and Ladouceur (2021) fail to acknowledge an important point: gambling disorder is a serious mental illness, with impaired control considered a central psychological construct of the condition (Dickerson, 2006). Once a gambler has developed a gambling disorder, in which impaired control is a central construct, it is insufficient for harm reduction strategies and the promotion of help-seeking to be largely passive, waiting for the individual with the gambling problem to actively seek help.

## Limitations

This literature review has some limitations which should be considered when interpreting these findings. Including only items published in English is a limitation that should be considered. While the search terms were broad and formulated through discussion with the research team and a research librarian, there may have been other useful terms not included which may have sourced additional articles. Additionally, despite conducting a wide-ranging systematic search, there is the possibility that some relevant articles were missed. A further limitation is that the quality of evidence included in the articles was not considered. Therefore, the findings are not conclusive but may provide some insight into where the research has focused to date, and areas that require further research.

## Conclusion

Despite the widely documented emphasis on venue staff to identify and respond to gamblers identified as having problems in situ, neither are staff inclined to approach them nor are gamblers to approach staff for assistance. Gambling-harm reduction strategies in venues should be multifaceted and consider and address all levels of influence on gambling behaviours. While health interventions are increasingly taking a population-health approach designed to address a range of levels, with a focus on primary prevention (Okechukwu et al., 2014), approaches to gambling harm remain largely individually focused. Land-based gambling environments can play an important part in influencing the behaviour of individuals with gambling problems and addressing gambling-related harm. A responsible gambling agenda that frames problem gambling largely as individual choice and, by implication, a personal responsibility, does not adequately consider the importance of social context and broader contextual aspects of problem gambling. Researchers and policymakers should consider opportunities to modify the gambling environment in a way that influences the gambler’s behaviour and minimises harm, for example, limiting opening hours of land-based gambling venues and modifying gambling products so they are less harmful. Further, a re-thinking of the role frontline gambling venue staff play in addressing problem gambling is required, such as advising staff to provide evidence-based harm minimisation information to all gamblers rather than only those identified as already experiencing harm. Finally, appropriate outcome measures of the effectiveness of in-venue harm minimisation strategies should be developed and incorporated with any initiatives implemented.

## Data Availability

Not applicable.
